# Status of lipid control in Bangladeshi subjects with type 2 diabetes mellitus on lipid-lowering drugs: a multicenter, facility-based, cross-sectional study

**DOI:** 10.1186/s12902-023-01522-z

**Published:** 2023-12-05

**Authors:** Shahjada Selim, Muhammad Shah Alam, Samir Kumar Talukder, Md Lutful Kabir, Abu Jar Gaffar, Md Ahamedul Kabir, Nusrat Zarin, Shahin Ibn Rahman, Md Masud Un Nabi, Marufa Mustari, Md Firoj Hossain, Ahmed Ifrad Bin Raunak, Md Azizul Hoque, Md Rashedul Islam, Farhana Akter, Mohammad Abdul Hannan, Mohammad Saifuddin, Md Asaduzzaman, Mohammad Motiur Rahman, Afsar Ahammed, Md Abdur Rafi, Mohammad Jahid Hasan, A. B. M. Kamrul-Hasan

**Affiliations:** 1 Department of Endocrinology, Bangabandhu Sheikh Mujib Medical University, Dhaka, 1000 Bangladesh; 2 Department of Medicine, Army Medical College Cumilla, Cumilla, Bangladesh; 3 Department of Endocrinology, Rangpur Medical College, Rangpur, Bangladesh; 4 Department of Pathology, Naogaon Medical College, Naogaon, Bangladesh; 5 Department of Endocrinology, TMSS Medical College, Bogura, Bangladesh; 6 Department of Endocrinology, Bangladesh Institute of Health Sciences, Dhaka, Bangladesh; 7 Department of Endocrinology, BIRDEM General Hospital, Dhaka, Bangladesh; 8 Department of Endocrinology, Rajshahi Medical College, Rajshahi, Bangladesh; 9 Department of Endocrinology, Mugda Medical College, Dhaka, Bangladesh; 10 Department of Orthopedic Surgery, Mugda Medical College & Hospital, Dhaka, Bangladesh; 11 Department of Endocrinology, Sher-E-Bangla Medical College, Barishal, Bangladesh; 12 Department of Neurology, BIRDEM General Hospital, Dhaka, Bangladesh; 13 Department of Endocrinology, Chittagong Medical College, Chittagong, Bangladesh; 14 Department of Endocrinology, North East Medical College, Sylhet, Bangladesh; 15 Department of Endocrinology, Dhaka Medical College, Dhaka, Bangladesh; 16 Department of Endocrinology, Shaheed Sheikh Abu Naser Specialized Hospital, Khulna, Bangladesh; 17 Department of Medicine, Rajshahi Medical College Hospital, Rajshahi, Bangladesh; 18 Department of Endocrinology, National Institute of Traumatology and Orthopaedic Rehabilitation (NITOR), Sher-E- Bangla Nagar, Dhaka, Bangladesh; 19 Pi Research Development Center, Dhaka, Bangladesh; 20 Tropical Disease and Health Research Center, Dhaka, 1100 Bangladesh; 21 Department of Endocrinology, Mymensingh Medical College, Mymensingh, Bangladesh

**Keywords:** Type 2 diabetes mellitus, Dyslipidemia, Hypercholesterolemia, Lipid-lowering agent, Lipid control, Glycemic control

## Abstract

**Background:**

Achievement of lipid targets is crucial in patients with type 2 diabetes mellitus (T2DM) to mitigate the risk of cardiovascular diseases (CVD). Data on lipid-control status among patients with T2DM in Bangladesh are scarce. This study was conducted to determine the lipid-control status among patients with T2DM who were on lipid-lowering drugs in the country.

**Methods:**

This cross-sectional study was conducted in the diabetes outpatient departments of several tertiary hospitals in Bangladesh from January 2022 to December 2022. Adults of both sexes diagnosed with T2DM for at least one year and were on the lipid-lowering drug(s) for a minimum of 3 months were included in the study by consecutive sampling. Patients’ data were collected by face-to-face interviews, and blood samples were collected for fasting lipid profile. The lipid target was set at < 200 mg/dL for total cholesterol (TC), < 150 mg/dL for triglyceride (TG), < 100 mg/dL for low-density lipoprotein cholesterol (LDL-C), > 40 mg/dL for high-density lipoprotein cholesterol (HDL-C), and < 160 mg/dL for non-HDL cholesterol (non-HDL-C).

**Result:**

Three thousand sixty patients (age 44.7 ± 13.3 years, female 57%) with T2DM were evaluated. Overall, almost 81% of the study subjects achieved the LDL-C target. Besides, TC, TG, HDL-C, and non-HDL-C targets were achieved by 40.8, 21.6, 66.3, and 44.1% of patients, respectively. However, all the lipid parameters were under control in only 8.8% of patients. Almost 77.6% of the patients with ischemic heart disease, 81.5% of patients with stroke, and 65% of patients with CKD had LDL levels < 70 mg/dL. Only 10.03% achieved the HbA1c target of < 7%. 7.4% of patients achieved both HbA1c < 7% and LDL < 100 mg/dL and 5% achieved both HbA1c < 7% and LDL < 70 mg/dL. Advanced age (aOR 0.97, 95% CI 0.96, 0.98, *p* < 0.001), longstanding T2DM (aOR 0.53, 95% CI 0.39, 0.72, *p* < 0.001), and non-statin therapy (aOR 0.25, 95% CI 0.16, 0.37, *p* < 0.001) were negatively associated with lipid control (LDL < 100 mg/dL) while using oral hypoglycemic drugs or insulin (aOR 2.01, 95% CI 1.45, 2.77, *p* < 0.001) and having cardiovascular comorbidity (aOR 3.92, 95% CI 3.00, 5.12, *p* < 0.001) were positively associated with lipid control.

**Conclusion:**

Though most patients with T2DM achieved their target LDL level, the prevalence of both glycemic and overall lipid control was low in our study despite lipid-lowering therapy.

## Introduction

Type 2 diabetes mellitus (T2DM) has recently become a major global health concern, especially in lower and middle-income countries. Globally, more than 537 million people are affected by T2DM, contributing to almost 11% of deaths annually [1, 2]. Cardiovascular diseases (CVD) dominate the causes of mortality in these patients, which is attributable to approximately two third of the total deaths of these patients [3, 4].

The typical pattern of dyslipidemia, often called ‘diabetic dyslipidemia,’ is characterized by elevated levels of triglycerides (TG), low high-density lipoprotein cholesterol (HDL-C), and normal to mildly elevated low-density lipoprotein cholesterol (LDL-C) [5, 6]. This pattern of lipid abnormality results from hepatic overproduction of TG-rich very-low-density lipoprotein (VLDL) particles and accelerated exchange of TG in VLDL for cholesteryl esters in HDL-C and LDL-C [7]. Dyslipidemia is common in patients with T2DM. Different reports suggest that more than two-thirds of the patients with T2DM have dyslipidemia [8–13]. Diabetic dyslipidemia is particularly prevalent in developing countries due to inadequate diagnosis and proper management [14]. Bangladesh is a lower-middle-income country in the Southeast Asian region. More than 13 million people in this country are affected by T2DM [15, 16]. A recent report suggests that more than 71% of males and 73% of females with T2DM in the country have dyslipidemia [17].

A body of evidence demonstrates that lipid-lowering therapy has a cardioprotective role in patients with T2DM. It is evidenced that intensive statin regimes reduce the risk of major vascular events such as myocardial infarction and stroke by 15%, without significant side effects. Moreover, LDL-C reduction of 1 mmol/L (38.67 mg/dL) results in approximately 23% reduction in CVD events. For these reasons, statins are considered first-line treatments for lipid control in many patients with T2DM, even at diagnosis. In those patients with multiple risk factors for CVD, high-dose statin is recommended [18].

However, despite using statins, achieving optimum lipid control among patients with T2DM is challenging. For example, a study conducted in China indicated that only half of the patients achieved LDL-C control with their lipid-lowering agent [8]. Similar findings were also reported from South Africa [12]. An analysis in Europe and Canada showed that the total cholesterol (TC) and LDL-C control rates were 48% and 55% in statin-using T2DM patients [19]. In this context, evaluating lipid control status among patients with T2DM who are on lipid-lowering drugs is necessary for optimal therapeutic outcomes. Hence, the present study aimed to evaluate the proportion of patients with lipid control and identify associated factors among individuals living with diabetes receiving outpatient care in Bangladesh from January 2022 to December 2022.

## Methods

### Study design and setting

The present cross-sectional study was conducted in the diabetes outpatient departments of several tertiary hospitals in Bangladesh from January 2022 to December 2022. The hospitals are located in divisional and district-level cities and cover all eight administrative divisions of the country. All clinics provide specialist and referral services to patients from the relevant catch-up areas by specialist endocrinologists.

All the patients who visited the diabetes outpatient departments of the hospitals within the study period were considered the study population. The sample size for the present study had been calculated from the following formula: n = z^2^p(1-p)/d^2^, where p = estimated prevalence and d = precision of error. Based on previous evidence of the prevalence of lipid control among patients with T2DM as 50% [19], we calculated the sample size as 384. However, we included a total sample of 3060 patients with T2DM for better statistical inference. Inclusion criteria for the present study were male and female patients aged ≥ 18 years, diagnosed with T2DM for at least one year, and were on a minimum of 3 months of lipid-lowering therapy. Pregnant and lactating women, patients with other types of DM, and those with familial dyslipidemias and uncorrected secondary causes of dyslipidemia were excluded. Patients with incomplete information were also excluded. The study included all the patients meeting the inclusion criteria; consecutive sampling was used.

### Data collection

Data was collected through face-to-face interviews, review of medical records, and clinical examinations. The interviews were carried out by attending physicians using a semi-structured questionnaire that included socio-demographic information, diabetes-related information, presence or absence of comorbidities and complications of diabetes, including ischemic heart disease (IHD), peripheral vascular disease (PVD), stroke, chronic kidney disease (CKD), diabetic neuropathy, and diabetic retinopathy. Information about comorbidities was retrieved from the medical records of the patients. Hypertension (HTN) was defined as systolic blood pressure ≥ 140 mmHg or diastolic blood pressure ≥ 90 mmHg and/or currently taking antihypertensive drugs and/or self-reported history of hypertension and antihypertensive drugs. Body mass index (BMI) was calculated by height and weight and categorized as underweight (< 18.5 kg/m^2^), normal (18.5–22.9 kg/m^2^), overweight (23–24.9 kg/m^2^), and obese (≥ 25 kg/m^2^) according to the World Health Organization cutoff values for the Asian population [17]. Types of glucose-lowering drugs (oral, insulin, or combination therapy) were also recorded. Details of lipid-lowering medications, including generic name, class, and dosage, were recorded.

The laboratory data included the fasting lipid profile, including total TC, TG, HDL-C, and LDL-C. Fasting blood samples were collected following an overnight (8 to 12 h) fasting, and lipids were measured, including direct measurement of LDL-C, using an automatic analyzer in the laboratory of the corresponding center. Hemoglobin A1c (HbA1c) was also measured in the laboratories using the methods certified by the National Glycohemoglobin Standardization Program.

### Outcome measures

The lipid control status of the patients with T2DM who were receiving lipid-lowering agents was the outcome measure of the present study. The primary outcome was to determine the proportion of patients with T2DM who achieved LDL-C levels < 100 mg/dL [18] The secondary outcomes of the study were to determine the proportion of patients with T2DM who achieved total cholesterol of less than 200 mg/dL, triglycerides of less than 150 mg/dL, LDL-C of less than 100 mg/dL, and non-HDL-C of less than 160 mg/dL [18]. The secondary outcomes also included the proportion of patients with atherosclerotic cardiovascular diseases (ASCVD that includes IHD, stroke, and PVD) and/or CKD who achieved LDL-C < 70 mg/dL, as well as determining the proportion of patients who achieved both HbA1c < 7% and LDL-C < 100 mg/dL and HbA1c < 7% and LDL-C < 70 mg/dL [18].

### Statistical analysis

All the statistical analyses were done using STATA version 17.0 (StataCorp. 2021. Stata Statistical Software: Release 17. College Station, TX: StataCorp LLC). Descriptive statistics like frequencies and percentages for categorical variables and means with standard deviations (SD) or medians with interquartile ranges (IQR) and percentiles for numerical variables were calculated. Finally, a multiple logistic regression model was constructed including all the potential covariates determined from review of existing literature to determine the factors associated with lipid control among the study subjects. A two-sided *p*-value of < 0.05 was considered to be statistically significant.

### Ethical considerations

The study received ethical permission from the institutional review board of Bangabandhu Sheikh Mujib Medical University, Shahbag, Dhaka, Bangladesh (Ref. no.: BSMMU/2021/4786–211; Date: 20 November 2021). Informed written consent was taken from the study subjects. The researcher ensured confidentiality by assigning a study number to each file to record clinical data on the datasheet without recording personal information. The study was conducted according to Good Clinical Practice and the Declaration of Helsinki.

## Result

### Sociodemographic characteristics

A total of 3060 patients with T2DM were included in the present study, with a mean age of 44.7 (SD 13.3) years. Almost one-third of these patients are aged between 41 to 50 years. Nearly 57% of the participants were female and hailed from urban areas (Table 1).

**Table 1:** Sociodemographic characteristics of the participants (*N* = 3,060)

Characteristic	Overall, n (%) or mean (SD)	Male, n (%) or mean (SD)	Female, n (%) or mean (SD)
**Sex**			
Male	1,302 (42.55)		
Female	1,758 (57.45)		
**Age (years)**	44.73 (13.34)	47.04 (13.17)	43.01 (13.21)
**Residence**			
Urban	1,759 (57.48)	710 (54.53)	1,049 (59.67)
Peri-urban	1,022 (33.40)	452 (34.72)	570 (32.42)
Rural	279 (9.12)	140 (10.75)	139 (7.91)
**BMI (kg/m**^**2**^**)**	27.18 (4.33)	27.05 (4.13)	27.28 (4.47)
**BMI category (kg/m**^**2**^**)**			
Underweight	6 (0.20)	3 (0.23)	3 (0.17)
Normal	194 (6.34)	105 (8.06)	89 (5.06)
Overweight	1,077 (35.20)	412 (31.64)	665 (37.83)
Obese	1,783 (58.27)	782 (60.06)	1,001 (56.94)
**Waist circumference (cm)**	94.81 (7.31)	95.02 (7.60)	94.66 (7.10)
**Abdominal obesity**	2,741 (89.58)	1,003 (77.04)	1,738 (98.86)
**Duration of diabetes (years)**	5.29 (4.79)	5.71 (5.00)	4.97 (4.61)
**Duration of diabetes category (years)**			
Up to 5 years	1,954 (63.86)	773 (59.37)	1,181 (67.18)
5–10 years	665 (21.73)	336 (25.81)	329 (18.71)
> 10 years	441 (14.41)	193 (14.82)	248 (14.11)
**Diabetes medication**			
No drug (only lifestyle modification)	331 (10.82)	98 (7.53)	233 (13.25)
OHA	1,505 (49.18)	685 (52.61)	820 (46.64)
OHA + Insulin	1,224 (40.00)	519 (39.86)	705 (40.10)
**Comorbidities**			
Hypertension	2,087 (68.20)	894 (68.66)	1,193 (67.86)
Hypothyroidism	447 (14.61)	94 (7.22)	353 (20.08)
Hyperthyroidism	130 (4.25)	24 (1.84)	106 (6.03)
Pulmonary diseases	728 (23.79)	242 (18.59)	486 (27.65)
Musculoskeletal diseases	733 (23.95)	233 (17.90)	500 (28.44)
Psychiatric diseases	821 (26.83)	267 (20.51)	554 (31.51)
**Complications of diabetes**			
Neuropathy	1,919 (62.71)	767 (58.91)	1,152 (65.53)
Nephropathy	1,202 (39.28)	450 (34.56)	752 (42.78)
Retinopathy	748 (24.44)	258 (19.82)	490 (27.87)
Ischemic heart disease	2,179 (71.21)	883 (67.82)	1,296 (73.72)
Stroke	745 (24.35)	262 (20.12)	483 (27.47)
Peripheral vascular disease	1252 (40.9)	533 (40.94)	719 (40.90)
**HbA1c (%)**	9.55 (2.30)	9.45 (2.23)	9.62 (2.35)
**HbA1c level**			
< 7%	307 (10.03)	137 (10.52)	170 (9.67)
7–8.9%	1,071 (35.00)	474 (36.41)	597 (33.96)
≥ 9%	1,682 (54.97)	691 (53.07)	991 (56.37)
**Lipid lowering drug**			
Moderate intensity statin (Atorvastatin 10–20 mg or Rosuvastatin 5–10 mg)	2,918 (95.36)	1,225 (94.09)	1,693 (96.30)
High-intensity statin (Atorvastatin 40–80 mg or Rosuvastatin 20–40 mg)	23 (0.75)	17 (1.31)	6 (0.34)
Non-statin (Fenofibrate)	119 (3.89)	60 (4.61)	59 (3.36)

*BMI* Body mass index, *OHA* Oral hypoglycemic agent

Most participants were overweight/obese (93.5%), with a mean BMI of 27.2 (SD 4.3); almost 89% had abdominal obesity. Almost 63.8% of the participants were suffering from T2DM for less than five years while almost 21.7% were suffering from 21.7%. Nearly half of the patients were on oral glucose-lowering drugs, 10% were on insulin, and 40% were on combined therapy of oral agents and insulin. 72.3% of them had comorbid diseases; HTN was the most common comorbidity, with a prevalence of 68.2% (Table 1).

Most (84.7%) patients had diabetic complications; IHD was present in 71.2%, diabetic neuropathy in 62.7%, PVD in 40.9%, and diabetic nephropathy in 39.3% of the participants. Most (90%) patients had poor glycemic control with a mean HbA1c level of 9.5% (SD 2.3). The majority (95.4%) of the patients were on moderate-intensity statin therapy (Atorvastatin 10–20 mg or Rosuvastatin 5–10 mg), only 0.75% were on high-intensity statin therapy (Atorvastatin 40–80 mg or Rosuvastatin 20–40 mg) while 3.9% were on non-statin therapy (Fenofibrate) (Table 1).

### Lipid control status

**Fig. 1:**
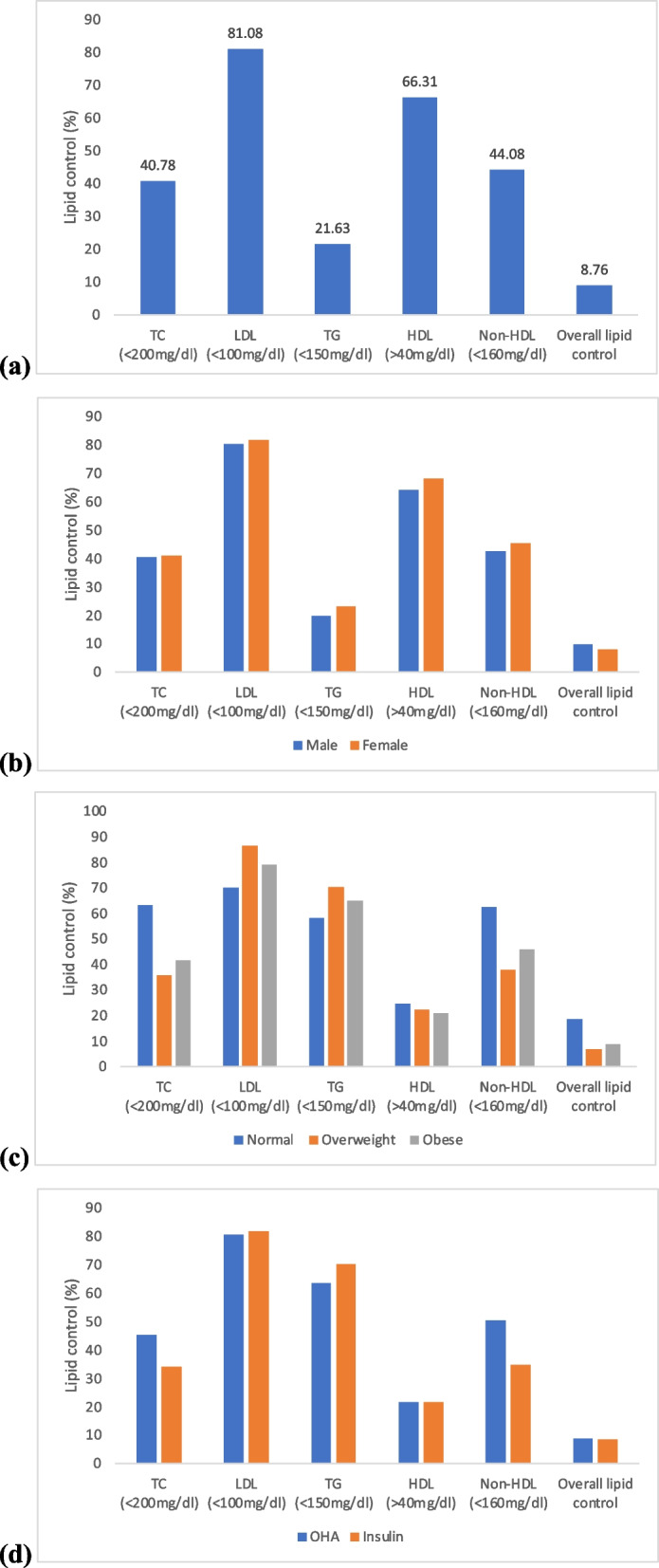
Lipid control status among patients with T2DM (**a** overall, **b** according to gender, **c** according to BMI, **d** according to hypoglycemic drugs)

Almost 81% of patients with T2DM achieved the LDL-C target of < 100 mg/dL. Besides, targets of TC < 200 mg/dL, TG < 150 mg/dL, HDL-C > 40 mg/dL, and non-HDL-C < 160 mg/dL were achieved by 40.8, 21.6, 66.3, and 44.1% of patients, respectively. However, all the lipid parameters were under control in almost 8.8% of patients (Fig. 1). Almost 77.6% of patients with IHD, 81.5% with stroke, 81.5% with PVD, and 65% with CKD had LDL-C levels < 70 mg/dL. Moreover, almost 66.7% of the patients from high-risk group (either having cardiovascular disease and/or chronic kidney disease) achieved the target of LDL-C levels < 70 mg/dL. Almost 7.4% of study subjects achieved both of the HbA1c target of < 7% as well as LDL-C target of < 100 mg/dL; 5% achieved HbA1c < 7% and LDL-C < 70 mg/dL.

### Factors associated with lipid control

In the logistic regression model, it was found that older age (aOR 0.97, 95% CI 0.96, 0.98, *p* < 0.001), longstanding T2DM (aOR 0.53, 95% CI 0.39, 0.72, *p* < 0.001) and non-statin therapy (aOR 0.25, 95% CI 0.16, 0.37, *p* < 0.001) were negatively associated with lipid control (LDL-C < 100 mg/dL). On the other hand, using oral hypoglycemic drugs or insulin (aOR 2.01, 95% CI 1.45, 2.77, *p* < 0.001) and having cardiovascular comorbidity (aOR 3.92, 95% CI 3.00, 5.12, *p* < 0.001) were positively associated with lipid control (LDL-C < 100 mg/dL) (Table 2).

**Table 2:** Factors associated with lipid control among patients with T2DM (Multiple logistic regression model)

Characteristic	LDL < 100 mg/dL	LDL ≥ 100 mg/dL	aOR (95% CI)	*p*-value
**Sex**				
Male	1,045 (80.26)	257 (19.74)	Ref	
Female	1,436 (81.68)	322 (18.32)	1.28 (0.63, 1.95)	0.150
**Age**^**a**^ (years), mean (SD)	43.55 (13.54)	49.77 (11.13)	0.97 (0.96, 0.98)	< 0.001
**BMI category**				
Underweight	4 (66.67)	2 (33.33)	Ref	
Normal	136 (70.10)	58 (29.90)	0.75 (0.10, 4.18)	0.8
Overweight	932 (86.54)	145 (13.46)	1.04 (0.14, 5.71)	> 0.9
Obese	1,409 (79.02)	374 (20.98)	0.79 (0.10, 4.34)	0.8
**Duration of diabetes category**				
Up to 5 years	1,691 (86.54)	263 (13.46)	Ref	
5–10 years	491 (73.83)	174 (26.17)	0.64 (0.49, 0.82)	< 0.001
> 10 years	299 (67.80)	142 (32.20)	0.53 (0.39, 0.72)	< 0.001
**Diabetes medication**				
No drug	237 (71.60)	94 (28.40)	Ref	
OHA	1,242 (82.52)	263 (17.48)	1.65 (1.21, 2.23)	0.001
OHA + Insulin	1,002 (81.86)	222 (18.14)	2.01 (1.45, 2.77)	< 0.001
**Cardiovascular comorbidity**				
**No**	177.00 (54.46)	148.00 (45.54)	Ref	
**Yes**	2,304 (84.24)	431 (15.76)	3.92 (3.00, 5.12)	< 0.001
**According to HbA1c level**				
< 7%	226 (73.62)	81 (26.38)	Ref	
≥ 7%	2255 (81.91)	498 (18.19)	1.12 (0.82, 1.54)	0.502
**Lipid lowering therapy**				
Moderate intensity statin	2,414 (82.73)	504 (17.27)	Ref	
High-intensity statin	17 (73.91)	6 (26.09)	1.05 (0.42, 3.02)	> 0.9
Non-statin (Fenofibrate)	50 (42.02)	69 (57.98)	0.25 (0.16, 0.37)	< 0.001

^a^Continuous variable

## Discussion

Attaining lipid targets is crucial in diabetes management, as dyslipidemia increases the risk of cardiovascular morbidity and mortality in these patients. Hence, lipid-lowering therapy is recommended as a preventive measure for patients with T2DM. Despite lipid-lowering therapy, achievement of lipid targets is a hefty task. The present study was conducted to determine lipid control among patients with T2DM in Bangladesh who were receiving lipid-lowering drugs.

In the present study, almost 81% of patients with T2DM who were receiving lipid-lowering drugs achieved their LDL-C target of < 100 mg/dL. Though achievement of the target for individual lipid components was comparatively high in this study, overall lipid control was observed in only 8.8% of the patients. Moreover, only 7.4% of the patients achieved the HbA1c target < 7% and LDL-C target < 100 mg/dL, and 5% achieved HbA1c level < 7% and LDL-C level < 70 mg/dL.

All the patients in our study were on lipid-lowering drugs, mostly statins, such as different doses of rosuvastatin and atorvastatin. Despite lipid-lowering therapy, most patients did not achieve optimum lipid control. This phenomenon among patients with T2DM is not unique to Bangladesh and has been observed in different studies with varying prevalence. A recent meta-analysis including 24 studies from 20 countries reported that almost 49% of patients with T2DM achieved the target for LDL-C, 58% for HDL-C, and 62% for TG control [17]. A study from India, the neighboring country of Bangladesh, reported that almost 41.5% of the patients with T2DM on lipid-lowering therapy achieved the target level of LDL-C [19]. A study from South Africa observed a similar phenomenon where almost 87% of the patients with T2DM who were on lipid-lowering agents did not achieve lipid control [20]. The primary goal of statin therapy for lipid management is to lower LDL-C levels as it significantly reduces the risk of CVD and mortality in patients with T2DM [21].

In our study, a good number of patients achieved LDL-C control. However, almost 19% of the patients had LDL-C above target and remained at risk of major CVD events despite statin therapy. A study conducted in China indicated that almost half of the patients achieved LDL-C control with their lipid-lowering agents [8]. Similar findings were also reported from South Africa [20]. An analysis in Europe and Canada showed that the TC and LDL-C control rates were 48% and 55% in T2DM patients using statins [22]. High TG and low HDL levels contributed to the predominant uncontrolled lipid profile in the present study. Isolated hypertriglyceridemia was attributable to almost 44% of the overall uncontrolled lipid profile. A similar pattern of lipid control was also observed in previous studies from different countries [8–10, 12, 20]. In patients with T2DM, insulin resistance and hyperglycemia lead to elevated levels of triglycerides, which culminates in the overproduction of glycerol-rich lipoproteins by the liver. Increasing TG-rich lipoproteins reduce HDL-C production and additional LDL-C production [23, 24]. On the other hand, high TG and low HDL-C can lead to insulin resistance, resulting in poor glycemic control, creating a vicious cycle.

The failure to achieve optimum lipid control despite lipid-lowering intervention might be associated with various factors.

In the present analysis, we found that advanced age, long-standing diabetes, and non-statin lipid-lowering therapy increased the risk of uncontrolled lipid status in patients with T2DM. Though we observed a comparatively lower prevalence of lipid control among male patients, it was not statistically significant. Most of the patients included in our study were overweight or obese, which might hinder lipid control, though it was not found significant in the present study [25]. Though in our study, the duration of T2DM had a positive association with uncontrolled lipid profile, the association of glycemic status was insignificant. However, in recent studies, HbA1c has been suggested to have a linear relationship with uncontrolled lipid profiles, and HbA1c could reflect cholesterol and LDL-C levels among patients with T2DM. A marked increase in total cholesterol and triglyceride levels and a decrease in HDL levels were observed in patients with poor glycemic control compared to patients with good glycemic control [26, 27].

The present study is one of the largest and most recent pieces of evidence to depict the characteristics of the lipid control status among patients with T2DM in Bangladesh. The findings might have significant public health implication. It might be hypothesized that, patients who were on lipid lowering agents in our study, were not conscious about their medication and lifestyle which in turn would increase the disease burden of CVD in near future. Adequate public health awareness programs should be designed to increase medication efficacy in these patients. However, our study had several limitations that would be worth mentioning. All the patients included in our study were on lipid-lowering agents. Hence, our findings might not represent the actual scenario of dyslipidemia among the overall patient population of T2DM. First, our analyses were confined to patients attending different tertiary health facilities. Second, we did not evaluate patients' adherence and duration of lipid-lowering therapy. Moreover, recall and social desirability bias are inherent limitations in such a context. Fourth, our defined target of LDL as < 100 mg/dL might be higher than some guidelines, suggesting a lower target of < 70 mg/dL. Fifth, uncontrolled lipid profile due to secondary drug causes, such as thiazide diuretics and beta-blockers, was not quantified and could not completely be ruled out as a confounding factor. Moreover, being a tertiary care facility-based study including subjects, most of whom had diabetic complications, it might represent a particular patient population at the highest CVD risk and with difficult-to-control lipid profiles, which limits the generalizability of the findings. Finally, because we used cross-sectional surveys, the changes observed reflect only the risk profiles of the diabetes population at the time of the individual surveys [28, 29].

## Conclusion

Our study evidenced that few patients with T2DM who were on lipid-lowering therapy achieved the ultimate target for both glycemic and lipid status. However, the majority of the patients achieved their target for LDL control. Uncontrolled lipid profile is mostly attributable to high TG and low HDL-C levels among patients with T2DM in Bangladesh despite having lipid-lowering therapy. Advanced age, duration of diabetes, and non-statin therapy were found to increase the risk of uncontrolled lipid profile among these patients. Our findings draw the attention of policymakers to increase accessibility to potent lipid-lowering agents as well as clinicians for adequate management of hyperlipidemia in patients living with diabetes.

## Data Availability

Patient-level data will be available on request from the corresponding author.

## Abbreviations

BMI: Body Mass Index
CKD: Chronic Kidney Disease
CVD: Cardiovascular Diseases
HDL-C: High-Density Lipoprotein Cholesterol
IQR: Interquartile Ranges
LDL-C: Low-Density Lipoprotein Cholesterol
T2DM: Type 2 Diabetes Mellitus
TC: Total Cholesterol
TG: Triglyceride
VLDL: Very-Low-Density Lipoprotein
